# Incorporation of perfluorohexyl-functionalised thiophenes into oligofluorene-truxenes: synthesis and physical properties

**DOI:** 10.3762/bjoc.9.141

**Published:** 2013-06-27

**Authors:** Neil Thomson, Alexander L Kanibolotsky, Joseph Cameron, Tell Tuttle, Neil J Findlay, Peter J Skabara

**Affiliations:** 1 WestCHEM, Department of Pure and Applied Chemistry, University of Strathclyde, 295 Cathedral Street, Glasgow, G1 1XL, U.K.

**Keywords:** oligomers, perfluorinated-materials, star-shaped molecules, synthesis, truxene

## Abstract

Oligofluorene-functionalised truxenes containing perfluorohexylthiophene units at the terminal positions on the arms were synthesised, and their optical and electrochemical properties were investigated to determine the effect that the perfluorohexylthiophene unit has on the HOMO and LUMO properties of the oligomers. By synthesising a molecule with longer oligofluorene arms the effects of the perfluorohexylthiophene unit on larger oligomers was explored. The effect of steric hindrance from the perfluorohexyl chain was also evaluated by altering the position of the chain on the thiophene moiety.

## Introduction

Star-shaped oligomers share a common central unit with multiple arms branching from the core [1]. The morphology and electronic properties of these oligomers often vary from the linear versions of the arms alone.

**Figure 1:**
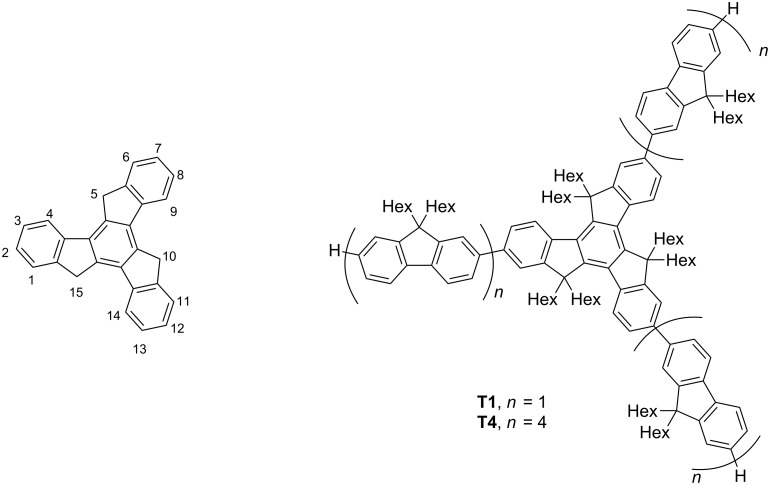
Labelled truxene and compounds **T1** and **T4**.

10,15-Dihydro-5*H*-diindeno[1,2-*a*;1′,2′-*c*]fluorene, also known as truxene (Figure 1), is a heptacyclic polyarene with *C_3_* symmetry that can be envisaged as three fluorenes that are superimposed through a common central benzene ring [2–3].

Truxene has been used as a starting compound, or a core unit, for larger star-shaped polyarenes such as fullerene fragments [4] and liquid-crystalline compounds [5]. Positions C5, C10 and C15 can be functionalised with a range of substituents, commonly saturated alkyl chains, which can enhance the solubility and processability as well as reduce intermolecular π–π stacking [6], but there are also examples of unsaturated alkyl [7] and phenyl [8] substituents at these positions. Cross-coupling reactions at the C2, C7 and C12 positions is the usual method of attaching “arms” to the core to form the star-shaped oligomers, such as **T1** and **T4** (Figure 1), extending the conjugation length and altering the photophysical properties of the resulting oligomer. There are also less common examples of functionalisation at the C3, C8 and C13 positions [9].

Alkyl substituents attached to hetero- and carbocyles, which make up the conjugated backbone of oligomers and polymers, can also affect the properties of the material [10]. Alkyl chains or other functional groups can donate or withdraw electron density from the polymer chain, for example by inductive effects, altering the HOMO–LUMO levels and subsequent band gap in the process. One type of substituent that can be used is a perfluoroalkyl chain, where the high percentage of fluorine present gives rise to a large electron-withdrawing effect [11]. However, perfluoroalkyl halides are not suitable alkylating reagents for S_N_2 type reactions, which makes the attachment of these types of chains to aromatic cyclic compounds difficult without the use of a spacer group, such as an ethylene chain, which can lessen the inductive effect [12]. Due to the widespread industrial use of polythiophenes, such as P3HT, analogous poly(perfluoroalkyl)thiophenes have received some interest due to their ability to affect certain properties of the polymer, such as the electrochemistry and stability, as well as the improved chemical resistance and thermal stability that replacing alkyl hydrogen atoms with fluorine atoms affords [13].

Our group has previously published work on oligofluorene-functionalised truxenes [14], and it was in our interest to observe changes in the optical and electrochemical properties by introducing perfluorohexylthiophene units at the terminal positions of the oligofluorene arms. Planarity of the backbone is an important concept in conjugated organic oligomers and polymers, as any twist in the dihedral angle between aromatic repeat units can lessen the p-orbital overlap, breaking the effective conjugation length [15]. This planarity can be perturbed by the steric effects of substituents attached to the hetero- or carbocycles in the conjugated backbone. In order to observe if the position of the perfluorohexyl chains would have an effect, two molecules that are structural isomers were synthesised and studied to compare the optical and electrochemical properties. Furthermore, a significantly larger molecule was synthesised to observe how a molecule with longer conjugated arms would also be affected by the perfluorohexylthiophene units.

## Results and Discussion

**Scheme 1:**
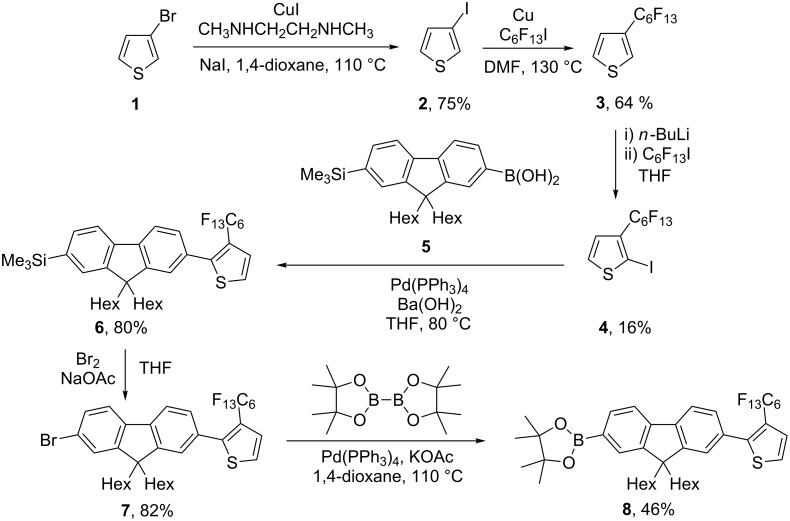
Synthesis of the thiophene-fluorene arm for the 3-isomer.

**Scheme 2:**
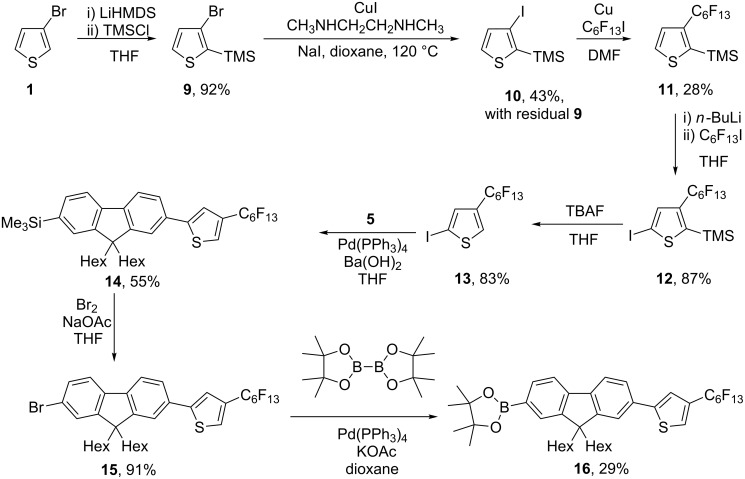
Synthesis of the thiophene-fluorene arm for the 4-isomer.

**Scheme 3:**
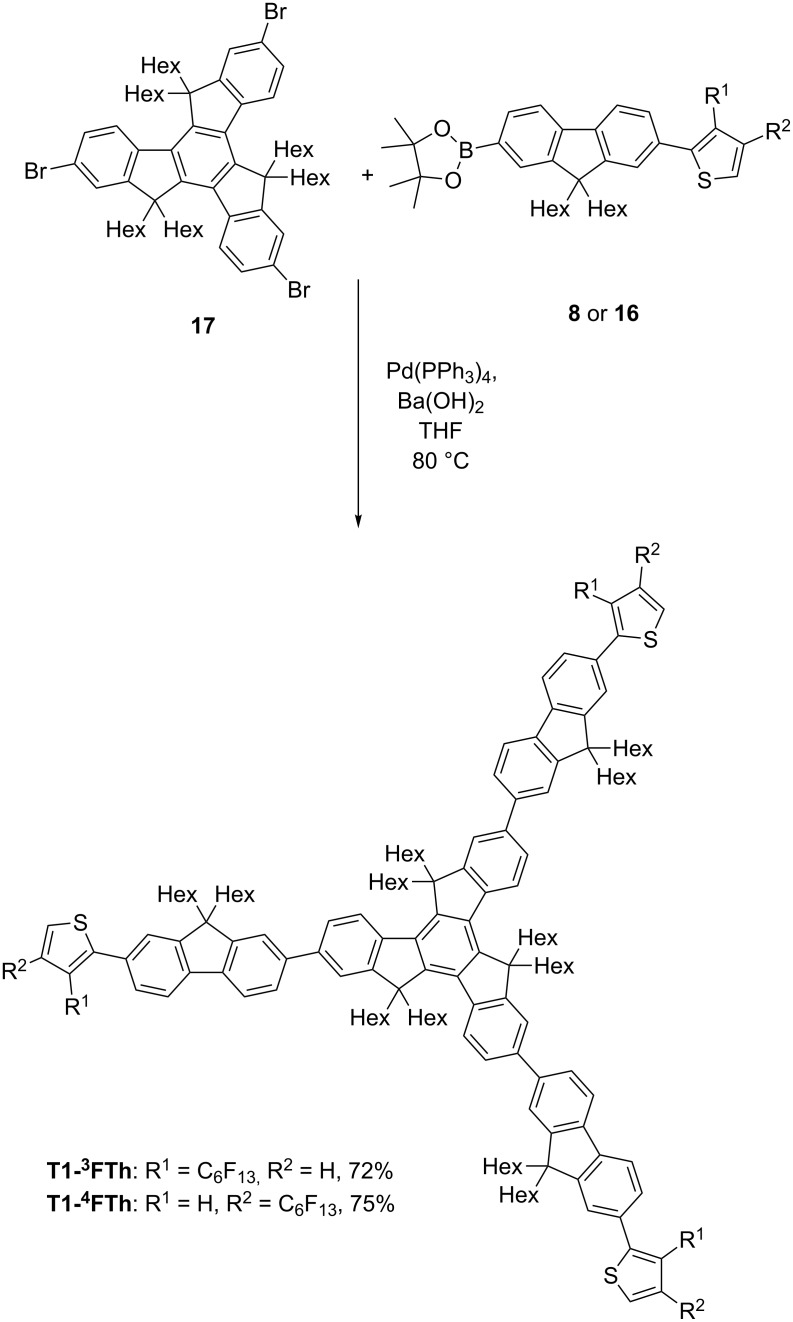
Coupling of arms to the truxene core.

**Scheme 4:**
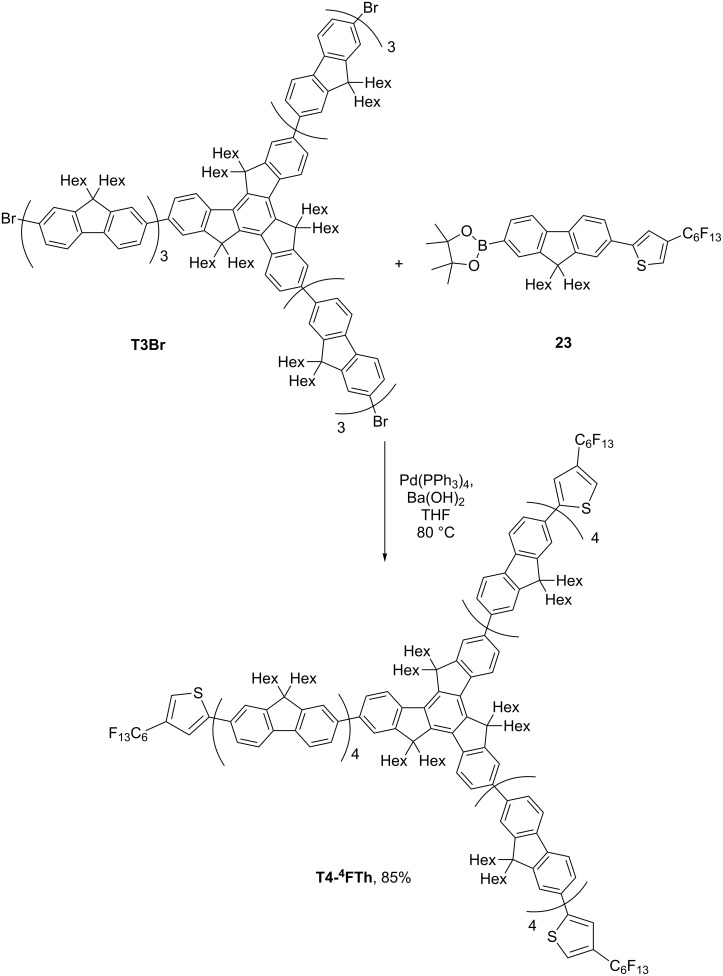
Synthesis of **T4-^4^FTh**.

The synthesis of the oligofluorene-functionalised truxenes is outlined in Schemes 1–4. 3-Perfluorohexylthiophene (**3**) was synthesised from 3-bromothiophene (**1**) by initial halogen exchange, using the method described by Buchwald et al. [16], to afford 3-iodothiophene (**2**), which then underwent a copper-mediated coupling reaction with perfluorohexyl iodide (Scheme 1). Lithiation of **3** and treatment with perfluorohexyl iodide gave the product 2-iodo-3-perfluorohexylthiophene (**4**). The regioselectivity of this reaction was poor, and the product was obtained as a mixture with its isomer, 2-iodo-4-perfluorohexylthiophene, which was separated by column chromatography. Compound **4** then underwent Suzuki–Miyaura coupling with compound **5**, which was synthesised according to our previously published method [17], and the product obtained, **6**, was deprotected by bromination to give compound **7** and then reacted with bis(pinacolato)diboron to afford boronic ester **8**.

The synthetic route to 2-iodo-4-perfluorohexylthiophene (**13**) (Scheme 2) began with treatment of 3-bromothiophene (**1**) with lithium bis(trimethylsilyl)amide and chlorotrimethylsilane to afford 2-trimethylsilyl-3-bromothiophene (**9**). However, although the conditions were unchanged, the halogen exchange reaction used to afford **10** was poorer yielding than that which produced **2**. This was due to incomplete conversion from the bromide to the iodide. The inability to separate **9** and **10** meant that this mixture was not further purified, and the mixture was taken forward to the next stage of the synthesis. The subsequent copper-mediated coupling reaction to afford **11** was also poor-yielding and a mixture of **9**, **10** and **11** was obtained. Compound **11** was obtained pure by a fluorinous extraction process with perfluorohexanes from a 5% water/ethanol solution. Lithiation and treatment with perfluorohexyliodide afforded **12**, which was deprotected with tetrabutylammonium fluoride to afford **13**. The boronic ester **16** was then synthesised by the same steps applied to obtain compound **8** (see Scheme 1).

**T1-^3^FTh** and **T1-^4^FTh** were then synthesised by Suzuki coupling of tribromohexahexyltruxene **17**, which was synthesised according to our previously published method [14], and the relevant boronic ester, **8** or **16** (Scheme 3).

**T4-^4^FTh** (Scheme 4) was synthesised by coupling compound **16** with **T3Br**. The latter compound was obtained by our previously published method [17].

**Figure 2:**
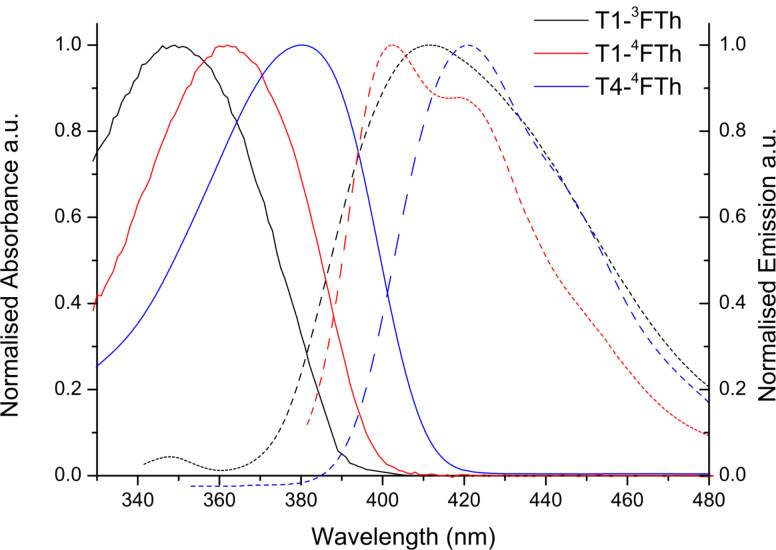
Normalised absorbance (solid) and emission (dashed) of materials in solution (dichloromethane).

The absorption and emission spectra of the oligomers in solution (dichloromethane), shown in Figure 2, all exhibit strong π–π^*^ transitions. The maximum absorption band was observed at 349 nm for **T1-^3^FTh**, 362 nm for **T1-^4^FTh**, and 380 nm for **T4-^4^FTh**. The maximum wavelength of emission was observed at 412 nm for **T1-^3^FTh**, 402 nm for **T1-^4^FTh**, and 421 nm for **T4-^4^FTh**. In comparison to the corresponding spectra of **T1** and **T4** (Table 1), the introduction of perfluorohexylthiophene units to the terminal positions on the arms leads to a red shift for both absorption and emission. The red shift within the **^4^FTh**-substituted series (**T1-^4^FTh** and **T4-^4^FTh**) increases with increasing chain length. The emission spectrum for **T1-^4^FTh** also shows a pronounced vibronic feature at 418 nm, which is an indication of the planarity of the molecular ground state in comparison to that of **T1-^3^FTh**, where no vibronic splitting is observed. The emission spectrum of **T4-^4^FTh** also reveals the vibronic structure**,** although it is less pronounced, with a shoulder at 440 nm. The Stokes shifts for **T1-^4^FTh** and **T4-^4^FTh** are 40 and 41 nm respectively; however, the shift for **T1-^3^FTh** is larger, at around 63 nm. This is an indication that the excited state of **T1-^3^FTh** undergoes a greater conformational change before emission than that of **T1-^4^FTh**, which is further evidence of the greater planarity in the **T1-^4^FTh** ground state compared to that of its isomer. The optical HOMO–LUMO gaps were estimated from the onset of absorption and were recorded as 3.20, 3.12 and 3.03 eV for **T1-^3^FTh**, **T1-^4^FTh** and **T4-^4^FTh**, respectively. The HOMO–LUMO gap for **T1-^3^FTh** and **T1-^4^FTh** is slightly narrower than that for **T1**; however, there is not much difference between the HOMO–LUMO gap of **T4-^4^FTh** and that of **T4**.

**Table 1:** Optical data for **TX-^x^FTh** and **TX** materials.

compound	λ_max_ (nm) absorption (CH_2_Cl_2_)	onset (nm)	ε (mM^−1^ cm^−1^)	HOMO–LUMO gap (eV)	λ_max_ (nm) emission (CH_2_Cl_2_)	Φ_PL_ (CH_2_Cl_2_)

**T1-^3^FTh**	349	387	306	3.20	412	—
**T1-^4^FTh**	362	397	323	3.12	402, 418	0.86
**T4-^4^FTh**	380	409	385	3.03	421, 440sh	0.93
**T1**^a^	343	—	—	3.29	375sh, 396, 416sh	—
**T4**^a^	374	—	—	3.05	411, 436, 460sh	—

^a^Values obtained from [14].

The results for the cyclic voltammetry of the oligomers are given in Table 2 (see Supporting Information File 1, Figures S1–S6 for the voltammograms). **T1-^3^FTh** showed one irreversible oxidation peak at +1.09 V, and a reduction peak was found at −2.22 V. From the onset of the oxidation and reduction peaks the HOMO level was estimated to be −5.72 eV and the LUMO −2.74 eV with reference to ferrocene, which was used as an internal standard and has a HOMO of −4.8 eV. In comparison to the HOMO and LUMO values for **T1** [14] (−5.6 and −2.2 eV) there is a decrease in the HOMO and a large decrease in the LUMO, with a reduction in the HOMO–LUMO gap. For **T1-^4^FTh**, quasi-reversible oxidation peaks were found at +0.86 and +1.03 V, while irreversible peaks were found as +1.26 and +1.44 V. A reduction peak was observed at −2.84 V. From the onset of the oxidation and reduction peaks the HOMO level was estimated to be −5.55 eV, and the LUMO −2.39 eV. Comparing these values to that of **T1**, the HOMO is essentially the same with the LUMO decreased, resulting in a narrower electrochemical band gap. A similar effect is observed for **T4-^4^FTh** in comparison with **T4** [14], where the HOMO is −5.53 eV compared to −5.5 eV for **T4** and the LUMO is lower (−2.35 eV for **T4-^4^FTh** and −2.3 eV for **T4**). The electrochemical HOMO–LUMO gaps for **T1-^3^FTh**, **T1-^4^FTh** and **T4-^4^FTh** were close to those derived from the absorption spectra, (within ca. 0.2 eV), which is in line with the differences observed for **T1** and **T4**.

**Table 2:** Electrochemical data for **TX-^X^FTh** and **TX**.

compound	*E*_ox_ (V) peaks *E*_Pa_/*E*_Pc_ or *E*_Pa_^a^	HOMO	*E*_red_ (V) peaks *E*_P_	LUMO	HOMO–LUMO gap (eV)

**T1-^3^FTh**	+1.09	−5.72	−2.22	−2.74	2.98
**T1-^4^FTh**	+0.86/0.79 +1.03/0.96 +1.26^b^ +1.44^b^	−5.55	−2.84	−2.39	3.16
**T4-^4^FTh**	+0.91/+0.82^c^ +1.11^c^	−5.53	−2.59	−2.35	3.18
**T1^d^**	—	−5.6	—	−2.2	3.4
**T4^d^**	—	−5.5	—	−2.3	3.2

^a^No cathodic peak is reported where the wave is irreversible. ^b^Peak exists as a shoulder. ^c^Two unresolved single-electron peaks. ^d^Values obtained from [14].

**Figure 3:**
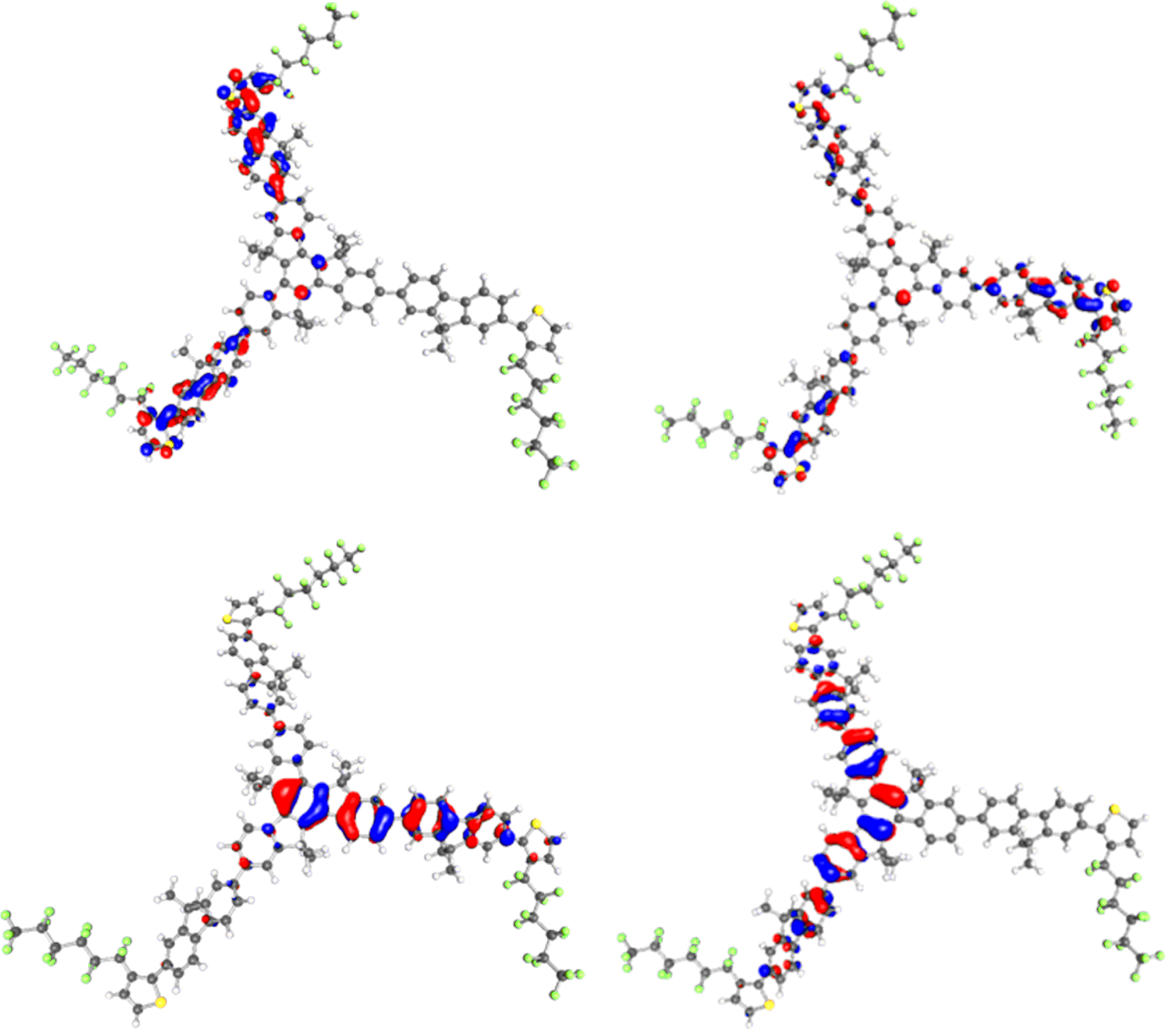
HOMO−1 (bottom, left), HOMO (bottom, right), LUMO (top, left) and LUMO+1 (top, right) of **T1-^3^FTh**.

**Figure 4:**
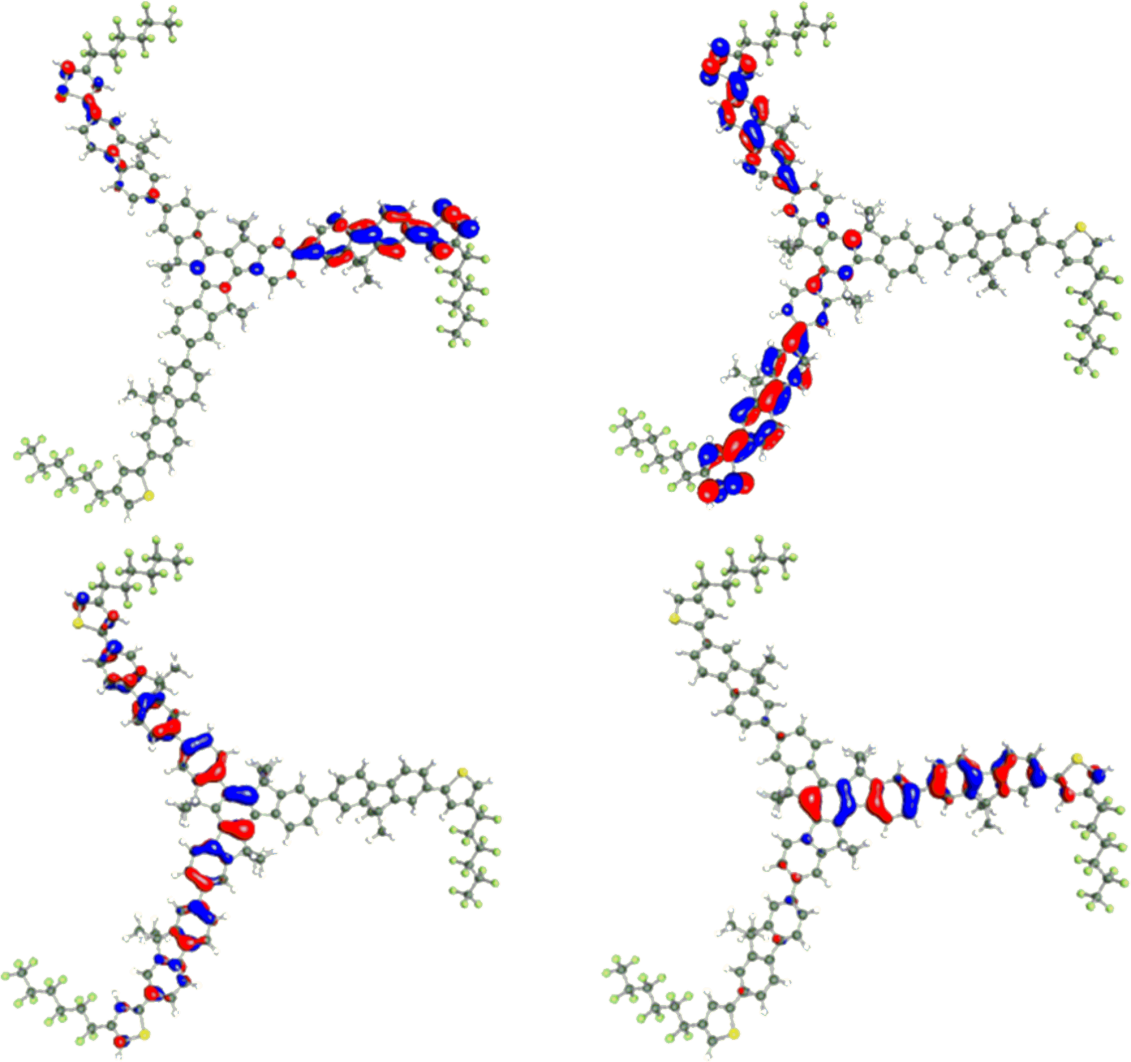
HOMO−1 (bottom, left), HOMO (bottom, right), LUMO (top, left) and LUMO+1 (top, right) of **T1-^4^FTh**.

In order to rationalise the difference in optical and structural properties between the two isomers, DFT calculations were performed on the smaller truxenes. The **T1-^3^FTh** and **T1-^4^FTh** structures were optimised in the gas phase by using the BP86-D [18–20] functional with the def2-TZVP [21] basis set implemented in TURBOMOLE 6.3.1 [22]. The optimisations were carried out by using the RI-J approximation [23]. The hexyl chains on the fluorene units were shortened to methyl groups in the model in order to decrease the computational cost of the simulation. The main structural difference observed is the degree of twisting between the fluorene and thiophene in the two structures. The average dihedral angle in **T1-^3^FTh** is 40.9°, whilst there is a much smaller twist of 6.4° in **T1-^4^FTh**. The frontier orbitals of the isomers were also compared and are shown below (Figure 3 and Figure 4). The HOMO−1 and HOMO for both molecules (Δ*E*[**T1-^3^FTh**] = 0.0054 eV and Δ*E*[**T1-^4^FTh**] = 0.0030 eV) are essentially degenerate and show a splitting due to a slight break in symmetry. This is also the case for the LUMO and LUMO+1 of each isomer (Δ*E*[**T1-^3^FTh**] = 0.013 eV and Δ*E*[**T1-^4^FTh**] = 0.0047 eV). The HOMO−1 and HOMO of the compounds show there to be a larger contribution of the thiophene unit to the conjugation of the molecule in **T1-^4^FTh** compared to **T1-^3^FTh**. This reaffirms that the thiophene unit of **T1-3FTh** is too twisted to contribute significantly to the conjugation of the molecule.

## Conclusion

In conclusion we have presented three new star-shaped oligomers, namely **T1-^3^FTh**, **T1-^4^FTh**, and **T4-^4^FTh**. For the smaller molecules, the presence of the powerfully electron-withdrawing perfluorohexyl chain within the molecules leads to a more stabilised LUMO, in comparison to the hydrogen-terminated molecule **T1**. The optical and electrochemical properties of the molecules with shorter oligofluorene arms are altered to a much greater extent than those of the molecules containing longer oligofluorene arms, where HOMO and LUMO levels as well as the optical HOMO–LUMO gaps remain essentially the same for the substituted and the parent systems. The electrochemical properties of **T1-^3^FTh** are more affected by the presence of the perfluoroalkyl thiophene substituent than those of **T1-^4^FTh**.

## Supporting Information

File 1 Experimental procedures and cyclic voltammetry details.
