# Supplementary material for: Incorporation of perfluorohexyl-functionalised thiophenes into oligofluorene-truxenes: synthesis and physical properties
Source: Beilstein J Org Chem. 2013 Jun 27;9:1243–51. doi: 10.3762/bjoc.9.141 (PMC3701379; doi:10.3762/bjoc.9.141)
Supplement: File 1 — Experimental procedures and cyclic voltammetry details. [file Beilstein_J_Org_Chem-09-1243-s001.pdf]

Supporting Information

for

Incorporation of perfluorohexyl-functionalised thiophenes into oligofluorene-truxenes: synthesis and physical properties

Neil Thomson, Alexander L. Kanibolotsky, Joseph Cameron, Tell Tuttle, Neil J. Findlay and Peter J. Skabara*

Address: WestCHEM, Department of Pure and Applied Chemistry, University of Strathclyde, 295 Cathedral Street, Glasgow, G1 1XL, U.K.

Email: Peter J. Skabara - peter.skabara@strath.ac.uk

* Corresponding author

Experimental procedures and cyclic voltammetry details

General

^{1}H NMR were run on either a Bruker DPX 400 or AV 500 spectrometer at 400 MHz or 500 MHz respectively, while ^{13}C NMR were run on either a Bruker DPX 400 or AV 500 spectrometer at 100 MHz or 125 MHz respectively. ^{19}F NMR were run on a Bruker AV 400 spectrometer at 376 MHz. Chemical shifts are in parts per million (ppm) referenced to the solvent peak.

Low-resolution mass spectroscopy was carried out at the University of Strathclyde using a Finnigan LCQ duo ESI, while high-resolution mass spectroscopy was carried out at the University of Glasgow on a JEOL High Resolution Mass Spectrometer using FAB.

All reagents were obtained from commercial suppliers. Solvents were purified using a Pure-Solv 400 Solvent Purification system. 1,4-Dioxane was purified by distillation over sodium metal at atmospheric pressure.

All glassware was oven dried to remove traces of moisture before use.

The measurements of oxidation and reduction potentials were performed on a CHI660A Electrochemical Workstation with *iR* compensation at 100 mV s^{-1}. Dichloromethane was used as the solvent, aqueous Ag/AgCl as the reference electrode, with platinum wire and a glassy carbon disk (Ø = 3 mm) as the counter and working electrodes, respectively. The solutions contained the substrate in a concentration of 5×10^{-4} M, together with *n*-BuN_4PF_6 (0.1 M) as the supporting electrolyte. All CV experiments were referred to the Fc/Fc^+ couple as an internal standard.

Absorption spectra were recorded using a ThermoSpectronic Unicam UV 300, whilst emission spectra were recorded using a Perkin Elmer LS45 Luminescence Spectrometer.

Perfluorohexanes were obtained as FC-72 from 3M.

3-Iodothiophene (2) [1]

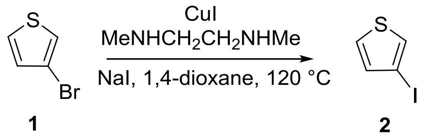

Copper iodide (0.71 g, 3.75 mmol, 0.05 equiv) and sodium iodide (22.5 g, 150 mmol, 2.0 equiv) were charged to the reaction vessel, which was backfilled with Ar.

3-Bromothiophene (**1**, 7 mL, 75.05 mmol, 1.0 equiv), *N*,*N'*-dimethylethylenediamine (0.74 mL, 7.5 mmol, 0.1 equiv) and 1,4-dioxane (80 mL) were added and the suspension heated at 110 °C under Ar for 24 h. The suspension was cooled to rt, diluted with hexane and filtered through silica gel (4 cm deep), eluting with diethyl ether. The solvent was then removed to afford the title product **2** as a yellow liquid (11.5 g, 75%).

The ^{1}H NMR of spectrum was recorded;

δ_H (400 MHz, $CDCl_3$): 7.42–7.41 (1H, dd, *J* = 3.0, 1.0 Hz, CH), 7.22–7.20 (1H, dd, *J* = 2.0, 3.0 Hz, CH), 7.13–7.11 (1H, dd, *J* = 5.0, 1.0 Hz, CH).

This is consistent with the previously published data [1].

3-Perfluorohexylthiophene (3) [2]

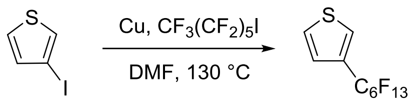

2 3

Copper powder (14.4 g, 228 mmol, 3.2 equiv) was suspended in dry DMF (100 mL) under Ar, to which 3-iodothiophene (**2**, 15 g, 71.4 mmol, 1.0 equiv) and perfluorohexyl iodide (23.2 mL, 107.1 mmol, 1.5 equiv) were added. The reaction mixture was heated at 130 °C for 20 h, after which time it was cooled and filtered through celite to afford a purple solution. The solution was poured into chilled conc. hydrochloric acid (75 mL acid, 75 g ice) and decanted. The solution was then extracted with hexane (4 × 250 mL). The organic layers were combined, washed with sodium sulphite solution (250 mL, 0.1 M) and water (250 mL), dried over magnesium sulphate and the solvent evaporated. The product was purified by silica gel chromatography eluting with hexane to afford the title product (**3**) as a yellow liquid (18.5 g, 64%).

The ^{1}H NMR, ^{19}F NMR and HRMS were recorded;

^{1}H NMR (400 MHz, $CDCl_3$): δ_H 7.75–7.74 (1H, m, CH), 7.47–7.44 (1H, m, CH), 7.26–7.24 (1H, m, CH); ^{19}F NMR (376 MHz, $CDCl_3$): δ_F −80.81 (3F), −106.07 (2F), −121.61 (2F), −122.26 (2F), −122.82 (2F), −126.12 (2F). HRMS (EI): found *m*/*z* 401.9752 (M^+), calcd for $C_{10}H_3F_{13}S$, 401.9748.

This was consistent with the previously published data [2].

2-Iodo-3-perfluorohexylthiophene (4)

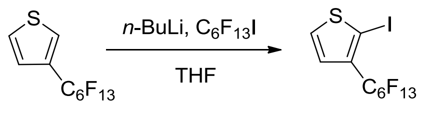

3-Perfluorohexylthiophene (**3**, 0.5 g, 1.24 mmol, 1.0 equiv) was dissolved in THF (40 mL) under Ar and cooled to −80 °C, to which *n*-butyllithium (2.4 M in hexanes, 0.57 mL, 1.36 mmol, 1.1 equiv) was added dropwise. The reaction mixture was allowed to warm to rt over 30 mins, then cooled again to −80 °C and perfluorohexyliodide (0.32 mL, 1.49 mmol, 1.2 equiv) was added, then allowed to warm to rt and stirred overnight under Ar. The reaction mixture was quenched with distilled water (100 mL) and the product was extracted with Et_2O (3 × 100 mL). The organics were combined, washed with sodium sulfite solution (1 M, 100 mL) and distilled water (100 mL), and dried over $MgSO_4$, and the solvent was evaporated. Kugelrohr distillation (ca. 80 °C) removed starting material, and the product was purified by silica gel chromatography eluting with hexane to afford the product **4** as a yellow oil (0.105 g, 16%).

^{1}H NMR (400 MHz, $CDCl_3$): δ_H 7.53 (1H, d, *J* = 5.6 Hz, CH), 6.99 (1H, d, *J* = 5.6 Hz, CH); ^{13}C NMR (100 MHz, $CDCl_3$): δ_C 133.5, 133.2, 128.5; ^{19}F NMR (376 MHz, $CDCl_3$): δ_F −80.76 (3F), −105.85 (2F), −120.66 (2F), −121.65 (2F), −122.74 (2F), −126.05 (2F).

(9,9-Dihexyl-7-(3-(perfluorohexyl)thiophen-2-yl)fluoren-2-yl)trimethylsilane (6)

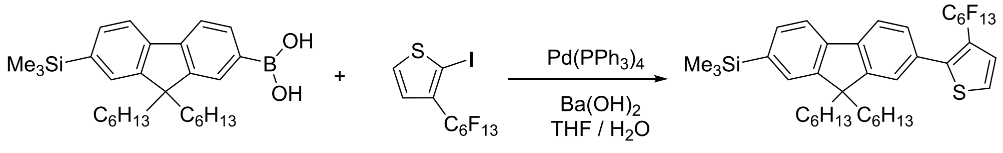

(9,9-Dihexyl-7-trimethylsilylfluoren-2-yl)boronic acid (**5**, 90 mg, 0.20 mmol, 1.05 equiv), 2-iodo-3-perfluorohexylthiophene (**4**, 100 mg, 0.19 mmol, 1.0 equiv), tetrakis(triphenylphosphine)palladium(0) (12 mg, 0.01 mmol, 0.05 equiv) and barium

hydroxide octahydrate (100 mg, 0.32 mmol, 1.6 equiv) were suspended in THF (8 mL) and distilled water (0.3 mL) under Ar, and heated at 80 °C for 20 h. The reaction mixture was cooled to rt, then quenched with saturated ammonium chloride solution (50 mL) and the product was extracted with CH_2Cl_2 (4 × 50 mL). The organics were combined, dried over $MgSO_4$ and the solvent was evaporated. The residue was purified by silica gel chromatography eluting with distilled petroleum ether and the product **6** was isolated as a yellow oil (0.12 g, 80%).

^{1}H NMR (400 MHz, $CDCl_3$): δ_H 7.71 (2H, d, *J* = 7.6 Hz, CH), 7.51 (1H, d, *J* = 7.6 Hz, CH), 7.48 (1H, s, CH), 7.40–7.37 (3H, m, CH), 7.22 (1H, d, *J* = 5.6 Hz, CH), 1.97–1.91 (4H, m, CH_2), 1.12–1.03 (12H, m, CH_2), 0.78–0.74 (6H, m, CH_3), 0.67–0.63 (4H, m, CH_2), 0.327 (9H, s, CH_3); ^{13}C NMR (100 MHz, $CDCl_3$): δ_C 150.8, 150.6, 149.2, 142.2, 141.3, 140.1, 132.2, 130.9, 129.3, 127.9, 127.6, 125.6, 125.2, 125.0, 119.6, 119.4, 119.3, 55.5, 40.5, 31.7, 31.7, 31.6, 29.9, 23.9, 22.81, 22.78, 22.75, 14.3, 14.21, 14.18; ^{19}F NMR (376 MHz, $CDCl_3$): δ_F −80.85 (3F), −102.05 (2F), −120.94 (2F), −121.80 (2F), −122.85 (2F), −126.16 (2F); *m*/*z* (ESI) 805.85 ($[M]^+$, 100%). HRMS (EI): found *m*/*z* 806.2642 (M^+), calcd for $C_{10}H_3F_{13}S$, 806.2647.

2-(7-Bromo-9,9-dihexylfluoren-2-yl)-3-(perfluorohexyl)thiophene (7)

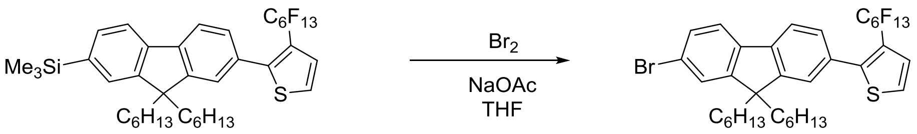

6 7

(9,9-Dihexyl-7-(3-(perfluorohexyl)thiophen-2-yl)fluoren-2-yl)trimethylsilane (**6**, 112 mg, 0.14 mmol, 1.0 equiv) and sodium acetate (11.5 mg, 0.14 mmol, 1.0 equiv) were dissolved in THF (7 mL) under Ar. The reaction vessel was covered in tin foil and cooled to 0 °C using an ice bath. Bromine (0.014 mL, 0.28 mmol, 2.30 equiv) was added to the reaction mixture dropwise, which was then stirred at 0 °C under Ar for 30 min. The reaction mixture was quenched with triethylamine (0.1 mL) followed by a sodium sulfite solution (1 M, 4 mL). The mixture was diluted with distilled water (10 mL) and the product was extracted with CH_2Cl_2 (5 × 20 mL). The organics were combined, washed with saturated sodium hydrogen carbonate solution (20 mL), then dried over $MgSO_4$ and the solvent was evaporated. The crude product was dissolved

in toluene and filtered through a silica plug, eluting with more toluene. The solvent was then evaporated to afford the product **7** as a yellow oil (0.094 g, 82%). This was used immediately without further purification.

^{1}H NMR (400 MHz, $CDCl_3$): δ_H 7.67 (1H, d, *J* = 8.4 Hz, CH), 7.59 (1H, d, *J* = 8.4 Hz, CH), 7.48 (2H, m, CH), 7.40–7.37 (3H, m, CH), 7.22 (1H, d, *J* = 5.6 Hz, CH), 1.97–1.93 (4H, m, CH_2), 1.12–1.03 (12H, m, CH_2), 0.78–0.75 (6H, m, CH_3), 0.67–0.63 (4H, m, CH_2); ^{13}C NMR (100 MHz, $CDCl_3$): δ_C 153.7, 150.3, 141.1, 139.8, 131.3, 130.5, 129.6, 127.7, 126.6, 125.7, 125.1, 122.0, 121.7, 119.5, 55.9, 40.7, 31.8, 29.9, 24.0, 22.9, 14.2; ^{19}F NMR (376 MHz, $CDCl_3$): δ_F −80.85 (3F), −102.05 (2F), −120.94 (2F), −121.77 (2F), −122.83 (2F), −126.14 (2F).

2-(9,9-Dihexyl-7-(3-(perfluorohexyl)thiophen-2-yl)fluoren-2-yl)-4,4,5,5-tetramethyl-1,3,2-dioxaborolane (8)

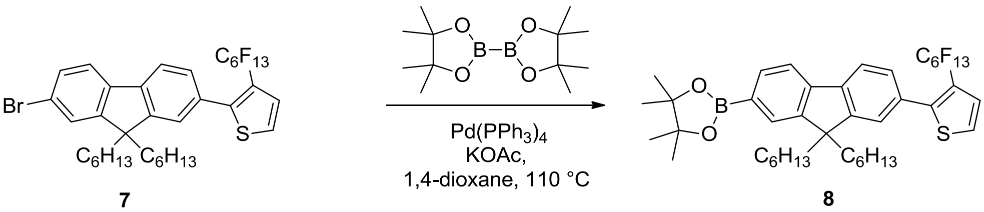

2-(7-Bromo-9,9-dihexylfluoren-2-yl)-3-(perfluorohexyl)thiophene (**7**, 550 mg, 0.68 mmol, 1.0 equiv), bis(pinacolato)diboron (208 mg, 0.82 mmol, 1.2 equiv), tetrakis(triphenylphosphine)palladium(0) (38 mg, 0.03 mmol, 0.05 equiv) and potassium acetate (186 mg, 1.90 mmol, 2.8 equiv) were dissolved in dry 1,4-dioxane (7 mL) under Ar, and heated at 110 °C for 20 h. The reaction mixture was cooled to rt and quenched with distilled water (50 mL), then the product was extracted with Et_2O (4 × 40 mL). The organics were combined, washed with brine (50 mL), and dried over $MgSO_4$, and the solvent was evaporated. The product was purified by silica gel chromatography eluting first with ethyl acetate/hexane 1:50 to remove impurities then with 1:20 to afford the product **8** as an orange paste (0.272 g, 46%).

^{1}H NMR (400 MHz, $CDCl_3$): δ_H 7.84–7.81 (1H, m, CH), 7.76–7.72 (2H, m, CH), 7.49–7.47 (1H, m, CH), 7.40–7.37 (3H, m, CH), 7.22 (1H, d, *J* = 5.6 Hz), 2.01–1.93 (4H, m, CH_2), 1.36 (12H, s, CH_3), 1.11–1.02 (12H, m, CH_2), 0.77–0.73 (6H, m, CH_3), 0.68–0.5 (4H, m, CH_2); ^{13}C NMR (100 MHz, $CDCl_3$): δ_C 150.2, 150.6, 149.1, 143.7,

142.0, 135.1, 134.2, 131.3, 139.3, 139.2, 128.1, 127.6, 125.6, 125.2, 119.8, 119.6, 84.1, 55.6, 40.6, 31.8, 30.0, 25.3, 25.2, 24.0, 22.9, 14.2; ^{19}F NMR (376 MHz, $CDCl_3$): δ_F −80.83 (3F), −102.08 (2F), −120.94 (2F), −121.79 (2F), −122.82 (2F), −126.14 (2F). *m*/*z* (ESI) 860.35 ($[M]^+$, 100%). HRMS (EI): found *m*/*z* 860.3104 (M^+), calcd for $C_{10}H_3F_{13}S$ 860.3116.

T1-^{3}FTh

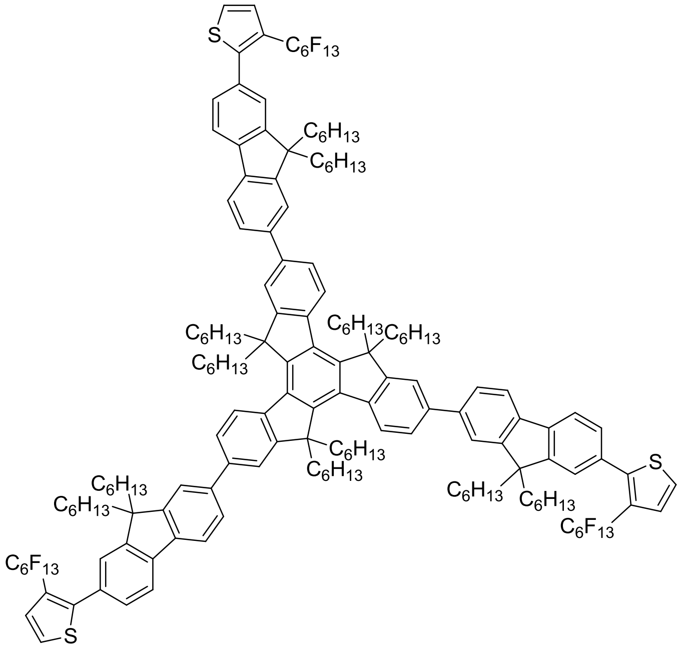

2-(9,9-Dihexyl-7-(3-(perfluorohexyl)thiophen-2-yl)fluoren-2-yl)-4,4,5,5-tetramethyl-1,3,2-dioxaborolane (**8**, 210 mg, 0.24 mmol, 4.77 equiv), tribromohexahexyltruxene **17** (54.2 mg, 0.05 mmol, 1.0 equiv), tetrakis(triphenylphosphine)palladium(0) (17.3 mg, 0.015 mmol, 0.3 equiv) and barium hydroxide octahydrate (115 mg, 0.37 mmol, 7.3 equiv) were suspended in THF (5 mL) under Ar, to which degassed distilled water (0.65 mL) was added. The reaction mixture was then heated at 80 °C for 18 h, then cooled to rt and quenched with a saturated aqueous solution of ammonium chloride (50 mL). The crude product was extracted with CH_2Cl_2 (6 × 50 mL), the organics were combined and dried over $MgSO_4$, the solvent was evaporated, and the crude product was placed under vacuum overnight. The product was loaded onto silica and eluted with a petroleum ether/toluene 20:1 mixture to afford the product as a white powder (110 mg, 72%).

^{1}H NMR (400 MHz, $CDCl_3$): δ_H 8.52 (3H, s, CH), 7.87 (3H, d, *J* = 7.6 Hz, CH), 7.81–7.76 (12H, m, CH), 7.53 (3H, d, *J* = 8.0 Hz, CH), 7.50–7.41 (9H, m, CH), 7.25 (3H, d, *J* = 5.2 Hz, CH) 3.08–3.07 (6H, m, CH_2) 2.28–2.26 (6H, m, CH_2), 2.10–2.08 (12H, m, CH_2), 1.14–1.07 (36H, m, CH_3), 0.98–0.87 (36H, m, CH_3), 0.80–0.77 (30H, m, CH_2), 0.66–0.62 (30H, m, CH_2); ^{13}C NMR (100 MHz, $CDCl_3$): δ_C 154.8, 152.3, 150.9, 149.2, 145.6, 142.0, 141.1, 140.1, 140.0, 139.8, 138.5, 130.8, 129.5, 127.7, 126.5, 125.6, 125.3, 125.2, 125.0, 121.6, 120.9, 120.7, 119.4, 56.3, 55.8, 40.9, 37.5, 31.9, 31.8, 30.0, 29.9, 24.4, 24.1, 22.9, 22.7, 14.2; ^{19}F NMR (376 MHz, $CDCl_3$): δ_F −80.83 (9F), −102.00 (6F), −120.889 (6F), −121.75 (6F), −122.81 (6F), −126.15 (6F); MALDI-TOF MS: *m*/*z* 2959.39 ([M-C_6H_{13}]$^+$, 100%), 2981.01 ([M-C_6H_{13}+Na], 70%), 3044.50 ([M]$^+$, 35%).

2-Trimethylsilyl-3-bromothiophene (9)

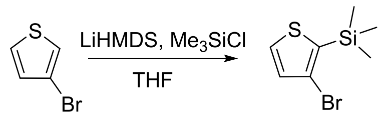

1 **9**

3-Bromothiophene (**1**), (5 mL, 53.4 mmol, 1.0 equiv) was dissolved in dry THF (50 mL) under Ar and cooled in an ice-bath, to which lithium bis(trimethylsilyl) amide (1.0 M in THF, 58.7 mL, 58.7 mmol, 1.1 equiv) was added dropwise. The reaction mixture was stirred for 1 h, after which time chlorotrimethylsilane (13.5 mL, 106.8 mmol, 2.0 equiv) was added dropwise and stirred for 2 h. The reaction mixture was quenched with sat. aq. ammonium chloride solution (200 mL) and distilled water (200 mL). The aqueous layer was extracted with Et_2O (350 mL), the organic layer was washed with water (200 mL) and dried over $MgSO_4$, and the solvent was evaporated. Kugelrohr distillation removed trace amounts of starting material **1**, and the crude product was purified by silica gel chromatography eluting with hexane to afford the product as a colourless oil (11.54 g, 92%).

The ^{1}H NMR spectrum was recorded;

^{1}H NMR (400 MHz, $CDCl_3$): δ_H 7.45 (1H, d, *J* = 4.8 Hz, CH), 7.11 (1H, d, *J* = 5.2 Hz, CH), 0.477 (9H, s, CH_3).

This was consistent with the previously published data [3].

2-Trimethylsilyl-3-iodothiophene (10)

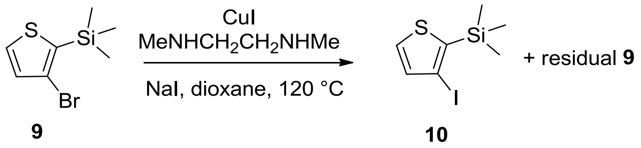

Copper iodide (0.45 g, 2.4 mmol, 0.05 equiv) and sodium iodide (14.65 g, 97.8 mmol, 2.0 equiv) were suspended in freshly distilled dioxane (75 mL) under Ar, to which 2-trimethylsilyl-3-bromothiophene (**9**, 11.5 g, 48.9 mmol, 1.0 equiv) and *N*,*N′*-dimethylethylenediamine (0.48 mL, 4.89 mmol, 0.1 equiv) were added. The reaction mixture was heated under reflux for 120 h, then cooled to rt. and filtered through a silica plug, washing with Et_2O. The solvent was removed to afford a mixture of the product **10** and starting material **9**. The crude material was used without further purification (6.5 g product, estimated by ^1H NMR integrals).

2-Trimethylsilyl-3-perfluorohexylthiophene (11)

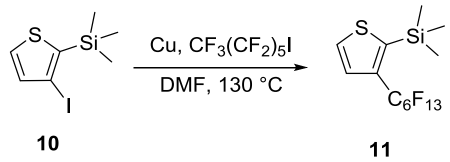

Copper powder (4.29 g, 68.03 mmol, 3.2 equiv) and a mixture of 2-trimethylsilyl-3-iodothiophene (**17**, 6.0 g, 21.26 mmol, 1.0 equiv) and 2-trimethylsilyl-3-bromothiophene (**10**) were suspended in dry DMF (50 mL) under Ar, to which perfluorohexyliodide (6.9 mL, 31.89 mmol, 1.5 equiv) was added. The reaction mixture was heated at 130 °C for 72 h, then cooled to rt, filtered over Celite and diluted with distilled water (100 mL). The aqueous layer was extracted with hexane (3 × 200 mL), then the organics were combined and washed with aq. sodium sulfite solution (1.0 M, 200 mL) and distilled water (100 mL) and dried over $MgSO_4$, and the solvent was evaporated. The crude product was suspended in a 5% H_2O/EtOH solution (30 mL), which was extracted with perfluorohexanes (2 × 25 mL). The fluorinated layer was dried over $MgSO_4$ and the solvent was evaporated to afford the product (**11**) as a yellow oil (2.85 g, 28%).

^{1}H NMR (400 MHz, $CDCl_3$): δ_H 7.63–7.61 (1H, m, CH), 7.34–7.32 (1H, m, CH), 0.38 (9H, s, CH_3); ^{13}C NMR (100 MHz, $CDCl_3$): δ_C 145.0, 134.7, 134.4, 131.3, 129.1, 0.57; ^{19}F NMR (376 MHz, $CDCl_3$): δ_F −80.85 (3F), −102.08 (2F), −120.21 (2F), −121.58 (2F), −122.80 (2F), −126.06 (2F); HRMS (EI): found *m/z* 474.0145 (M^+), calcd for $C_{10}H_3F_{13}S$, 474.0143.

2-Trimethylsilyl-3-perfluorohexyl-5-iodothiophene (12)

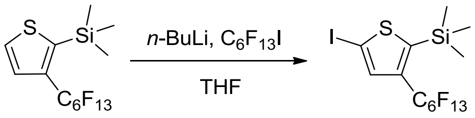

11 **12**

2-Trimethylsilyl-3-perfluorohexylthiophene (**11**, 2.5 g, 5.27 mmol, 1.0 equiv) was dissolved in dry THF (100 mL) under Ar and cooled to −80 °C, to which *n*-butyllithium (2.37 M in hexanes, 2.44 mL, 5.80 mmol, 1.1 equiv) was added dropwise and stirred for 15 min at −80 °C, after which perfluorohexyliodide (1.37 mL, 6.32 mmol, 1.2 equiv) was added dropwise. The reaction mixture was allowed to warm to rt and was stirred for 18 h, then quenched with sat. sodium hydrogen carbonate solution (100 mL) and diluted with distilled water (100 mL). The aqueous layer was extracted with Et_2O (3 × 150 mL), and the organic layers were combined and dried over $MgSO_4$, and the solvent was evaporated. The product was purified by filtering through a silica plug, eluting with hexane, to afford the product **12** as a golden oil (2.75 g, 87%).

^{1}H NMR (400 MHz, $CDCl_3$): δ_H 7.41 (1H, t, J = 1.6 Hz, CH), 0.37 (9H, s, CH_3); ^{13}C NMR (100 MHz, $CDCl_3$): δ_C 152.6, 138.3, 136.1, 79.0, 0.4; ^{19}F NMR (376 MHz, $CDCl_3$): δ_F −80.80 (3F), −102.77 (2F), −120.13 (2F), −121.55 (2F), −122.76 (2F), −126.04 (2F); HRMS (EI): found *m/z* 599.9107 (M^+), calcd for $C_{10}H_3F_{13}S$, 599.9110.

2-Iodo-4-perfluorohexylthiophene (13)

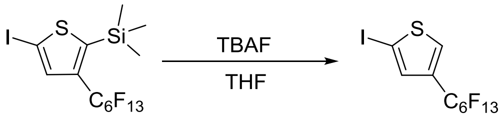

12 **13**

2-Trimethylsilyl-3-perfluorohexyl-5-iodothiophene (**12**, 2.75 g, 4.58 mmol, 1.0 equiv) was dissolved in dry (THF) under Ar, to which tetrabutylammonium fluoride (1.0 M, 4.81 mL, 4.81 mmol, 1.05 equiv) was added dropwise and stirred at rt for 4 h. The reaction mixture was quenched with distilled water (100 mL) and the aqueous layer was extracted with Et_2O (2 × 100 mL). The organics were combined and dried over $MgSO_4$, and the solvent was evaporated to afford a black oil. The crude product was filtered through a silica plug eluting with Et_2O to afford the product (**13**) as a brown oil (2.01 g, 83%).

^{1}H NMR (400 MHz, $CDCl_3$): δ_H 7.74 (1H, s, CH), 7.37 (1H, s, CH); ^{13}C NMR (100 MHz, $CDCl_3$): δ_C 135.0, 134.4, 132.1, 75.6; ^{19}F NMR (376 MHz, $CDCl_3$): δ_F −80.78 (3F), −106.68 (2F), −12.54 (2F), −121.07 (2F), −122.78 (2F), −126.02 (2F); HRMS (EI): found *m*/*z* 527.8710 (M^+), calcd for $C_{10}H_3F_{13}S$, 527.8714.

(9,9-Dihexyl-7-(4-(perfluorohexyl)thiophen-2-yl)-9*H*-fluoren-2-yl)trimethylsilane (14)

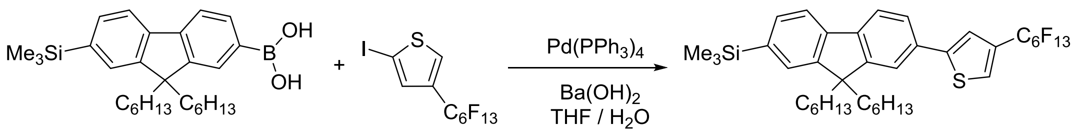

5 **13** **14**

9,9-Dihexyl-7-trimethylsilylfluoren-2-ylboronic acid (**5**, 1.34 g, 2.98 mmol, 1.05 equiv), 2-iodo-4-perfluorohexylthiophene (**13**, 1.5 g, 2.84 mmol, 1.0 equiv), tetrakis(triphenylphosphine)palladium(0) (0.16 g, 0.14 mmol, 0.05 equiv) and barium hydroxide octahydrate (1.46 g, 4.54 mmol, 1.6 equiv) were suspended in dry THF (120 mL) and distilled water (5 mL) under Ar, and heated at 80 °C for 20 h. The reaction mixture was cooled to rt then quenched with saturated ammonium chloride solution (200 mL), and the product was extracted with CH_2Cl_2 (4 × 200 mL). The organics were combined and dried over $MgSO_4$, and the solvent was evaporated. The product was purified by silica gel chromatography eluting with distilled petroleum ether and the product **14** was isolated as a yellow oil (1.25 g, 55%).

^{1}H NMR (400 MHz, $CDCl_3$): δ_H 7.74–7.69 (2H, m, CH), 7.66 (1H, s, CH), 7.59–7.57 (1H, m, CH), 7.54–7.50 (2H, m, CH), 7.48 (1H, s, CH), 7.44 (1H, s, CH), 2.03–1.99 (4H, m, CH_2), 1.14–1.07 (12H, m, CH_2), 0.78–0.75 (6H, t, *J* = 6.8 Hz, CH_3), 0.70–

0.66 (4H, m, CH_2), 0.33 (9H, s, CH_3); ^{13}C NMR (100 MHz, $CDCl_3$): δ_C 152.4, 150.4, 147.9, 142.1, 141.2, 140.1, 132.3, 132.2, 131.1, 128.0, 127.0, 125.4, 120.9, 120.7, 119.5, 55.6, 40.4, 31.7, 29.9, 24.0, 22.8, 14.3; ^{19}F NMR (376 MHz, $CDCl_3$): δ_F −80.75 (3F), −106.57 (2F), −121.46 (2F), −121.91 (2F), −122.72 (2F), −126.02 (2F); HRMS (EI): found *m*/*z* 806.2646 (M^+), calcd for $C_{10}H_3F_{13}S$, 806.2647.

2-(7-Bromo-9,9-dihexyl-9*H*-fluoren-2-yl)-4-(perfluorohexyl)thiophene (15)

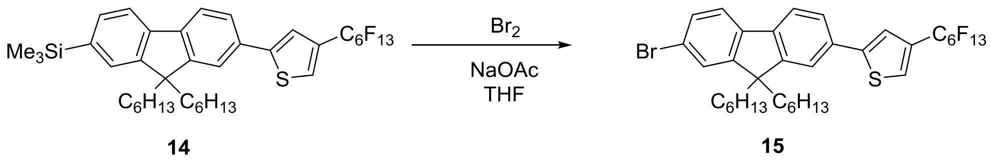

(9,9-Dihexyl-7-(4-(perfluorohexyl)thiophen-2-yl)-9*H*-fluoren-2-yl)trimethylsilane (**14**, 1.25 g, 1.55 mmol, 1.0 equiv) and sodium acetate (0.127 g, 1.55 mmol, 1.0 equiv) were dissolved in dry THF (50 mL) under Ar. The reaction vessel was covered in tin foil and cooled to 0 °C using an ice bath. Bromine (0.15 mL, 3.10 mmol, 2.0 equiv) was added to the reaction mixture dropwise, which was then stirred at 0 °C under Ar for 30 mins. The reaction mixture was quenched with triethylamine (1 mL) followed by a sodium sulfite solution (1 M, 50 mL). The mixture was diluted with distilled water (100 mL) and the product was extracted with CH_2Cl_2 (4 × 200 mL). The organics were combined, washed with saturated sodium hydrogen carbonate solution (100 mL), then dried over $MgSO_4$ and the solvent was evaporated. The crude product was dissolved in toluene and filtered through a silica plug, eluting with more toluene. The solvent was then evaporated to afford the product **15** as a yellow oil (1.15 g, 91%).

^{1}H NMR (400 MHz, $CDCl_3$): δ_H 7.70–7.67 (2H, m, CH), 7.61–7.57 (2H, m, CH), 7.52 (1H, d, *J* = 1.2 Hz, CH), 7.50–7.48 (2H, m, CH), 7.44 (1H, s, CH), 2.02–1.97 (4H, m, CH_2), 1.16–1.04 (12H, m, CH_2), 0.80–0.76 (6H, t, *J* = 6.8 Hz, CH_3), 0.68–0.62 (4H, m, CH_2); ^{13}C NMR (100 MHz, $CDCl_3$): δ_C 153.5, 151.8, 147.5, 141.0, 139.7, 132.5, 131.1, 130.5, 129.4, 128.6, 127.1, 126.6, 125.6, 121.9, 121.6, 121.1, 120.7, 120.6, 56.0, 40.6, 31.8, 30.0, 24.0, 22.9, 14.3; ^{19}F NMR (376 MHz, $CDCl_3$): δ_F −80.77 (3F), −106.58 (2F), −121.46 (2F), −121.90 (2F), −122.74 (2F), −126.00 (2F); HRMS (EI): found *m*/*z* 812.1347 (M^+), calcd for $C_{10}H_3F_{13}S$, 812.1357.

2-(9,9-Dihexyl-7-(4-(perfluorohexyl)thiophen-2-yl)-9*H*-fluoren-2-yl)-4,4,5,5-tetramethyl-1,3,2-dioxaborolane (16)

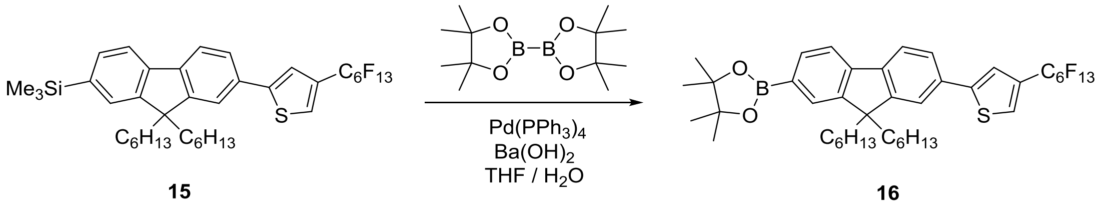

2-(7-bromo-9,9-dihexyl-9*H*-fluoren-2-yl)-4-(perfluorohexyl)thiophene (**15**, 1.15 g, 1.41 mmol, 1.0 equiv), bis(pinacolato)diboron (0.43 g, 1.69 mmol, 1.2 equiv), tetrakis(triphenylphosphine)palladium(0) (81 mg, 0.07 mmol, 0.05 equiv) and potassium acetate (0.39 g, 3.95 mmol, 2.8 equiv) were dissolved in dry dioxane (14 mL) under Ar, and heated at 110 °C for 20 h. The reaction mixture was cooled to rt and quenched with distilled water (100 mL), then the product was extracted with Et_2O (4 × 100 mL). The organics were combined, washed with brine (100 mL), and dried over $MgSO_4$, and the solvent was evaporated. The product was purified by silica gel chromatography eluting first with ethyl acetate/hexane 1:50 to remove impurities then with 1:20 to afford the product **16** as an brown oil (0.35 g, 28%).

^{1}H NMR (400 MHz, $CDCl_3$): δ_H 7.86–7.84 (1H, d, *J* = 7.6 Hz, CH), 7.79–7.71 (3H, m, CH), 7.67 (1H, d, CH), 7.61–7.59 (1H, m, CH), 7.53 (1H, s, CH), 7.46 (1H, s, CH), 2.08–2.01 (4H, m, CH_2), 1.41(12H, s, CH_3) 1.14–1.02 (12H, m, CH_2), 0.78–0.75 (6H, t, *J* = 6.8 Hz, CH_3), 0.69–0.62 (4H, m, CH_2); ^{13}C NMR (100 MHz, $CDCl_3$): δ_C 152.8, 150.48, 147.8, 143.6, 142.0, 134.3, 132.5, 131.1, 129.3, 129.2, 127.1, 125.4, 121.0, 120.6, 119.6, 84.2, 55.7, 41.6, 31.8, 30.0, 25.3, 24.0, 22.9, 14.3; ^{19}F NMR (376 MHz, $CDCl_3$): δ_F −80.79 (3F), −106.58 (2F), −121.47 (2F), −121.91 (2F), −122.74 (2F), −126.05 (2F); HRMS (EI): found *m*/*z* 860.3099 (M^+), calcd for $C_{10}H_3F_{13}S$, 860.3116

T1-^{4}FTh

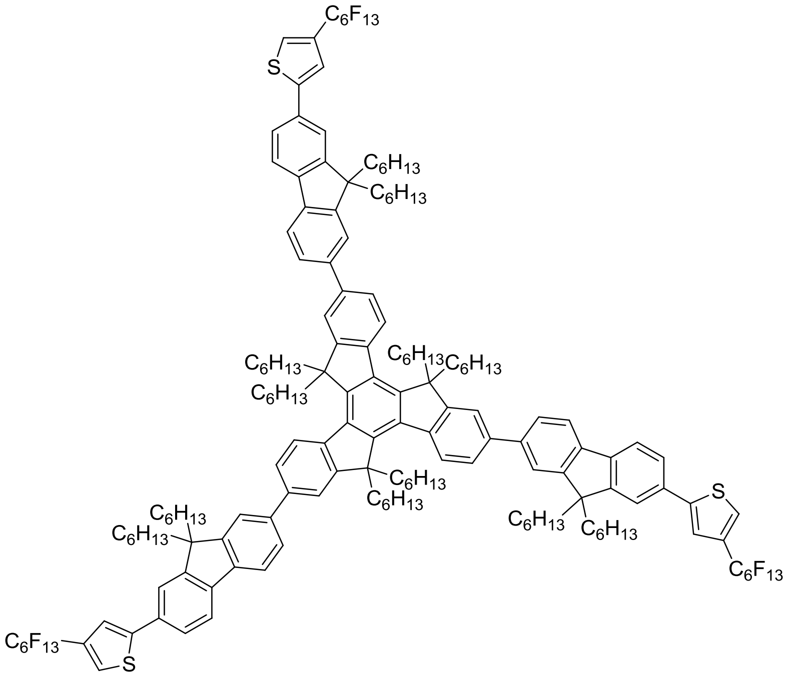

2-(9,9-dihexyl-7-(4-(perfluorohexyl)thiophen-2-yl)-9*H*-fluoren-2-yl)-4,4,5,5-tetramethyl-1,3,2-dioxaborolane (**16**, 350 mg, 0.407 mmol, 4.77 equiv), tribromohexahexyltruxene **17** (92 mg, 0.085 mmol, 1.0 equiv), tetrakis(triphenylphosphine)palladium(0) (29 mg, 0.0255 mmol, 0.3 equiv) and barium hydroxide octahydrate (196 mg, 0.62 mmol, 7.3 equiv) were suspended in THF (8 mL) under Ar, to which degassed distilled water (1 mL) was added. The reaction mixture was then heated at 80 °C for 18 h, then cooled to rt and quenched with a saturated aqueous solution of ammonium chloride (50 mL). The crude product was extracted with CH_2Cl_2 (6 × 50 mL), the organics were combined and dried over $MgSO_4$, the solvent was evaporated, and the crude product was placed under vacuum overnight. The product was loaded onto silica and eluted with a petroleum ether/toluene 15:1 mixture to afford the product as a sticky yellow solid (195 mg, 75%). ^{1}H NMR (400 MHz, $CDCl_3$): δ_H 8.52 (3H, d, *J* = 8.4 Hz, CH), 7.86 (3H, d, *J* = 8.0 Hz, CH),7.80 (12H, d, *J* = 6.4 Hz, CH), 7.76 (3H, s, CH), 7.69 (3H, s, CH), 7.64 (3H, d, *J* = 8.0 Hz, CH), 7.60 (3H, s, CH) 7.48 (3H, s, CH) 3.10–3.08 (6H, m, CH_2), 2.27–2.24 (6H, m, CH_2), 2.14–2.11 (12H, m, CH_2), 1.17–1.11 (36H, m, CH_2), 1.00–0.92 (36H, m, CH_3), 0.81–0.78 (30H, m, CH_2), 0.69–0.62 (30H, m, CH_2); ^{13}C NMR (100 MHz, $CDCl_3$): δ_C 154.8, 152.5, 152.1, 147.8, 145.6, 141.9, 141.0, 140.1, 140.0, 139.8, 138.5, 132.1, 131.1, 127.0, 126.6, 125.6, 125.3, 121., 121.0, 120.6, 56.3,

55.9, 40.8, 37.5, 31.9, 31.8, 30.0, 29.9, 24.4, 24.2, 22.86, 22.69, 22.7, 14.32, 14.27, 14.2; ^{19}F NMR (376 MHz, $CDCl_3$): δ_F −80.73 (3F), −106.57 (2F), −121.46 (2F), −121.90 (2F), −122.73 (2F), −126.03 (2F); MALDI-TOF MS: *m/z* 2959.39 ([M-C_6H_{13}]$^+$, 100%), 2981.01 ([M-C_6H_{13}+Na], 70%), 3044.50 ([M]$^+$, 35%).

T4-^{4}FTh

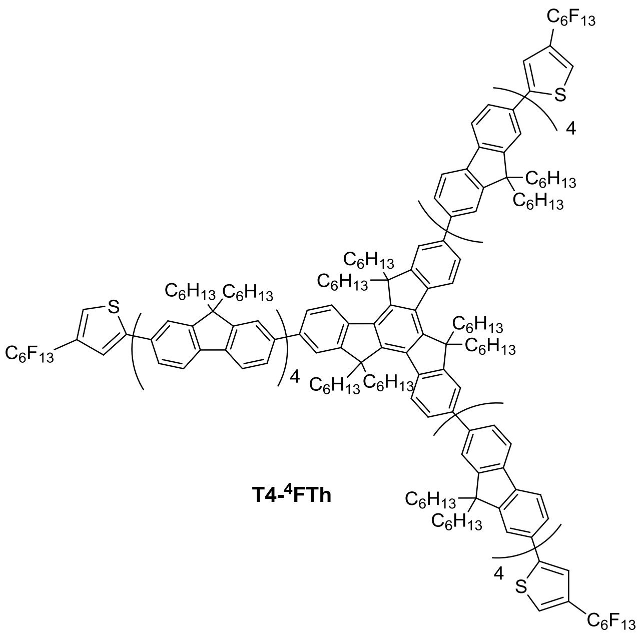

T4-^{4}FTh

The mixture of tribromterfluoreneotruxene core compound (**T3-3Br**, 46,1 mg, 11.3 μmol), tetrakis(triphenylphosphine)palladium(0) (16.5 mg, 14.3 μmol), 2-(9,9-dihexyl-7-(4-(perfluorohexyl)thiophen-2-yl)-9*H*-fluoren-2-yl)-4,4,5,5-tetramethyl-1,3,2-dioxaborolane (**16**, 54.0 mg, 62.7 μmol), $Ba(OH)_2\cdot 8H_2O$ (58.7 mg, 186 μmol) in 5 mL of THF and 0.12 mL of water was heated under reflux for 24 h at 80 °C. After aqueous work-up the crude product was subjected to the column on silica, eluting with CH_2Cl_2/PE 1:7. Re-precipitation from CH_2Cl_2/methanol afforded 57.9 mg of the product (0.959 μmol, 85%) as an off-white amorphous solid.

^{1}H NMR (400 MHz, $CDCl_3$) δ_H, 8.70–8.40 (1H, d, broad), 8.00–7.56 (27H, m), 7.47 (1H, s), 3.30–2.90 (2H, s, broad), 2.48–1.90 (18H, m), 1.30–0.60 (110H, m); ^{13}C NMR ($CDCl_3$, δ, 100 MHz) 154.62, 152.28, 151.99, 151.89, 147.63, 145.38, 141.64, 141.24, 140.78, 140.74, 140.51, 140.33, 140.28, 140.22, 140.13, 139.81, 139.70, 139.64, 138.35, 131.84, 130.89, 126,45, 126.35, 125.35, 121.71, 121.50, 120.70, 120.44, 56.05, 55.61, 55.55, 55.51, 40.51, 31.37, 31.74, 31.66, 31.63, 29.89,

29.83, 29.77, 24.25, 24.03, 23.94, 22.75, 22.72, 22.51, 14.18, 14.12, 14.10; ^{19}F NMR (376 MHz, $CDCl_3$): δ_F −80.73 (3F), −106.56 (2F), −121.45 (2F), −121.88 (2F), −122.71 (2F), −126.01 (2F); MALDI-TOF MS: *m/z* 6037(M^+), 5952($[M–C_6H_{13}]^+$). Anal. calcd for $C_{393}H_{477}F_{39}S_3$: C, 78.17; H, 7.96; found: C, 77.92; H, 7.85.

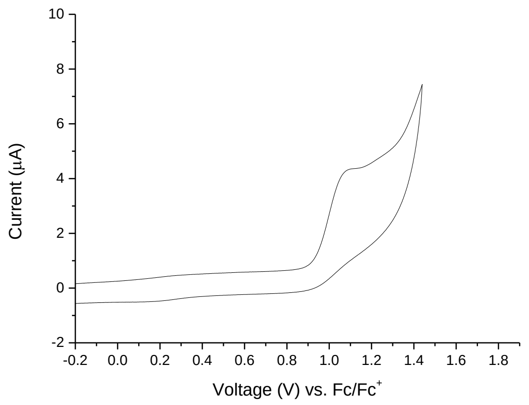

Figure S1: Oxidation wave from cyclic voltammetry of **T1-^{3}FTh** in dichloromethane, electrolyte 0.1 M Bu_4NPF_6, glassy carbon electrode, scan rate 100 mV s^{-1}.

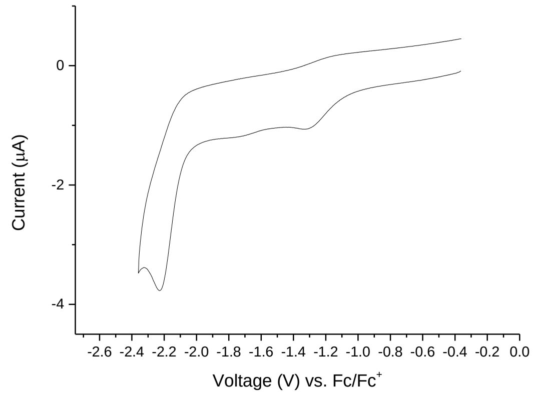

Figure S2: Reduction wave from cyclic voltammetry of **T1-^{3}FTh** in tetrahydrofuran, electrolyte 0.1 M Bu_4NPF_6, glassy carbon electrode, scan rate 100 mV s^{-1}.

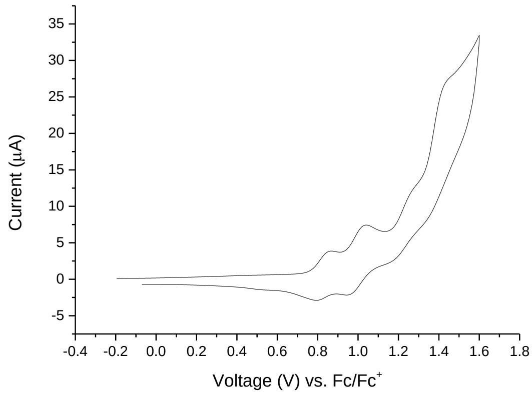

Figure S3: Oxidation wave from cyclic voltammetry of **T1-^{4}FTh** in dichloromethane, electrolyte 0.1 M Bu_4NPF_6, glassy carbon electrode, scan rate 100 mV s^{-1}.

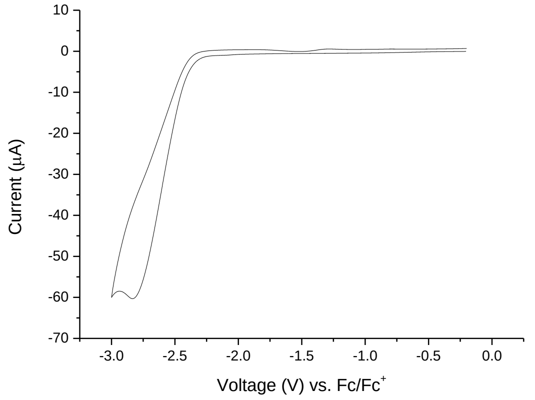

Figure S4: Reduction wave from cyclic voltammetry of **T1-^{4}FTh** in tetrahydrofuran, electrolyte 0.1 M Bu_4NPF_6, glassy carbon electrode, scan rate 100 mV s^{-1}.

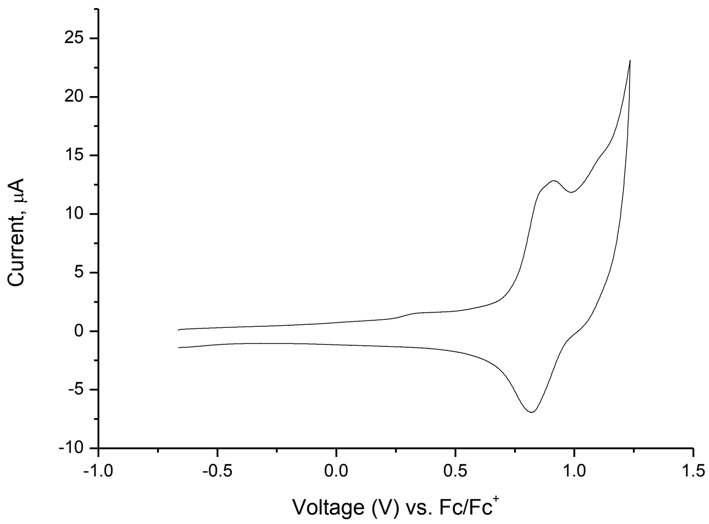

Figure S5: Oxidation wave from cyclic voltammetry of **T4-[4]FTh** in 1:1 acetonitrile/benzene, electrolyte 0.1 M Bu_4NPF_6, glassy carbon electrode, scan rate 100 mV s^{-1}.

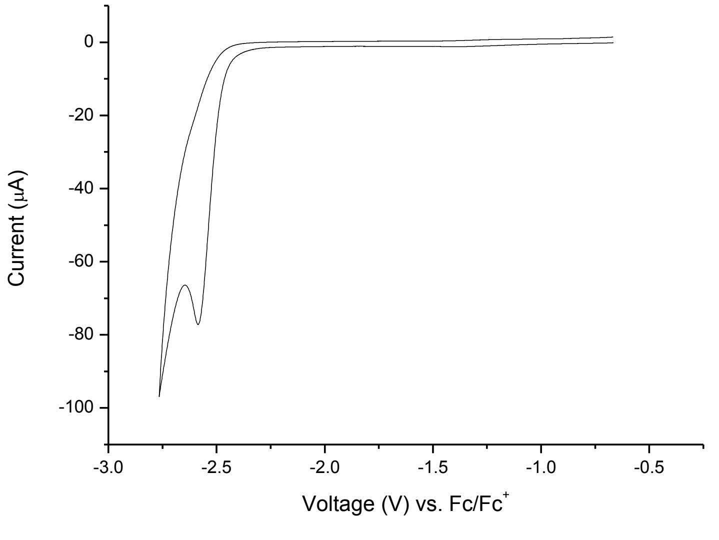

Figure S6: Reduction wave from cyclic voltammetry of **T4-[4]FTh** in 1:1 acetonitrile/benzene, electrolyte 0.1 M Bu_4NPF_6, glassy carbon electrode, scan rate 100 mV s^{-1}.

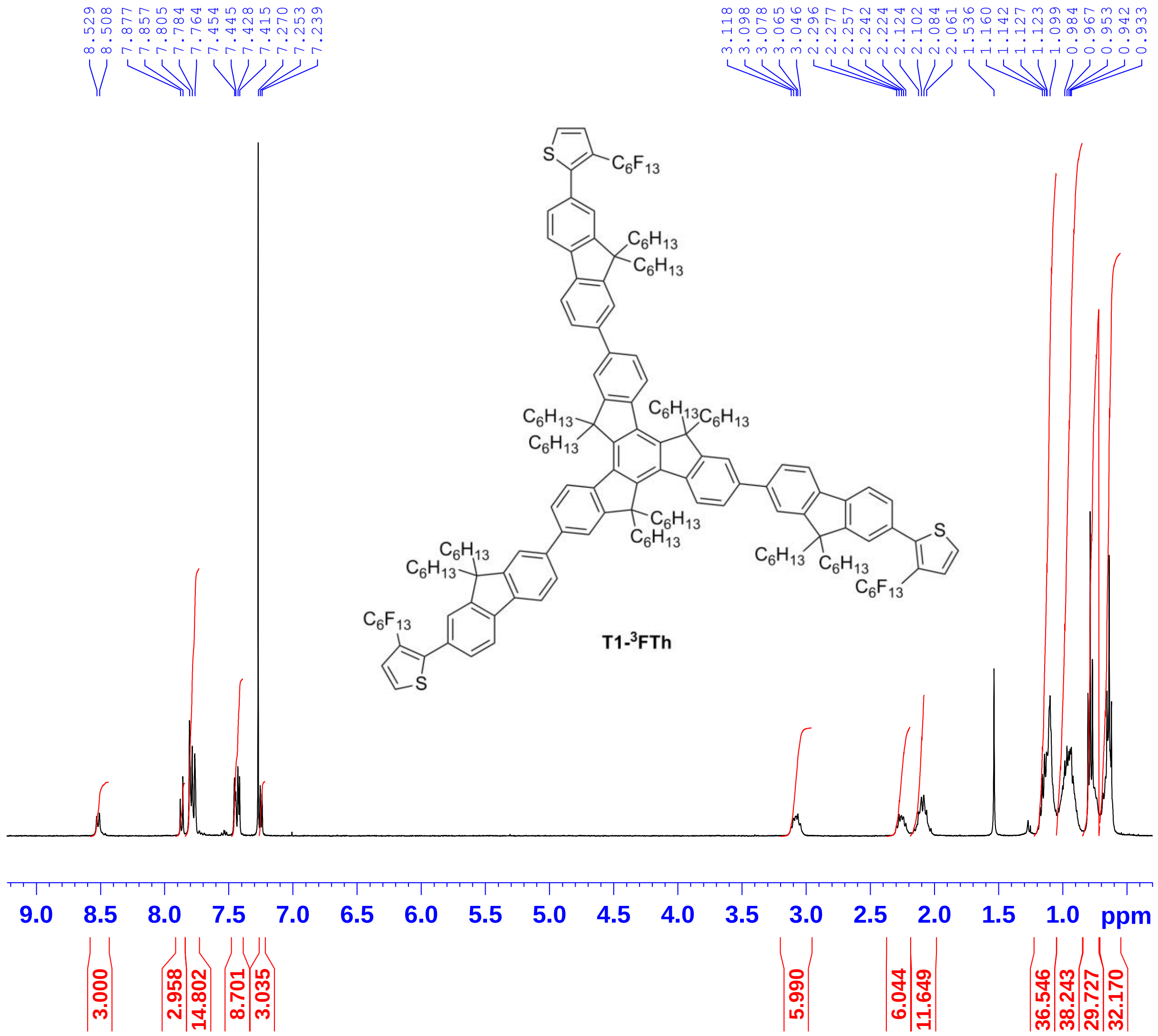

Figure S7: ^{1}H NMR of **T1-^{3}FTh**.

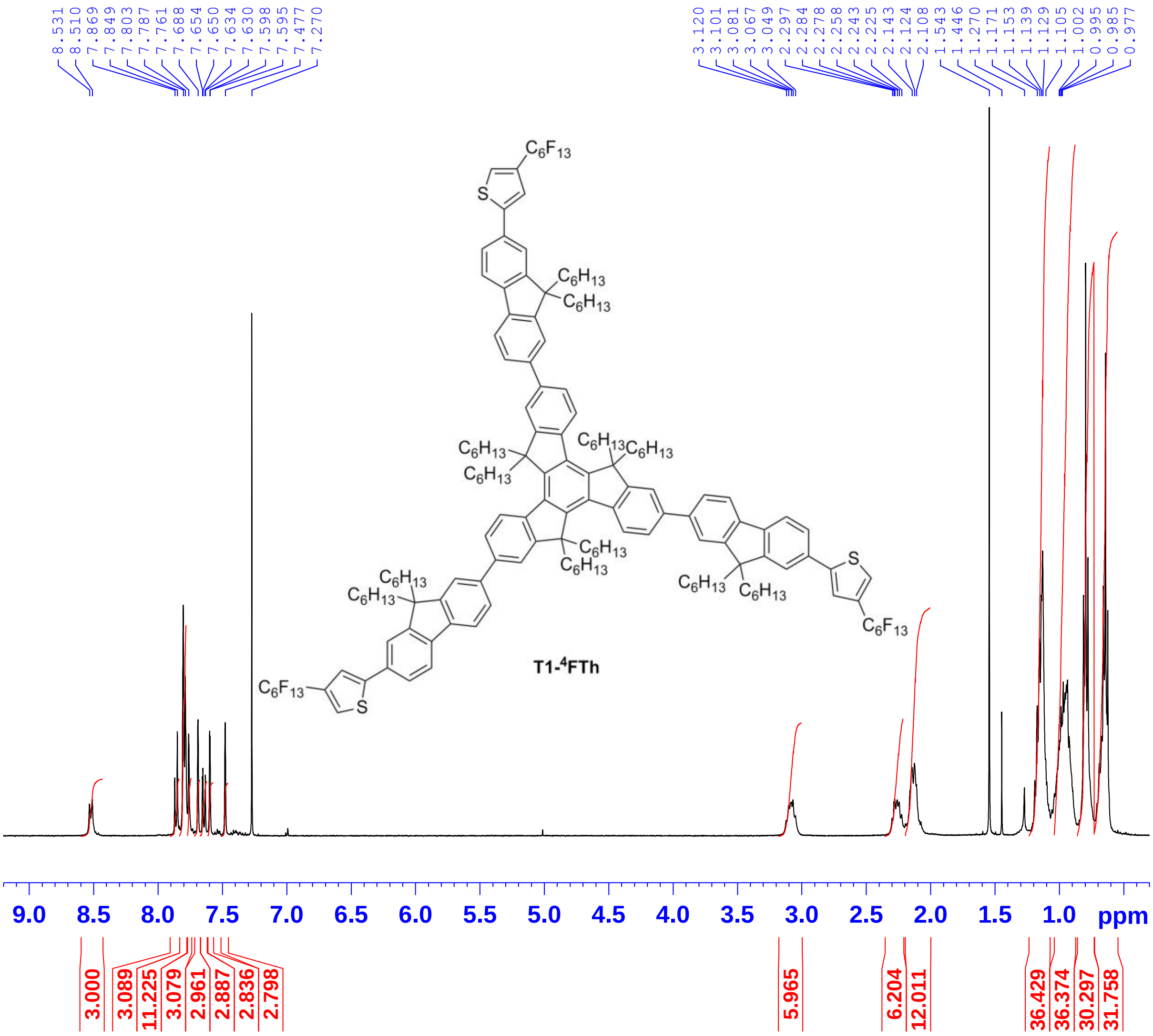

Figure S8: ^{1}H NMR of **T1-^{4}FTh**.

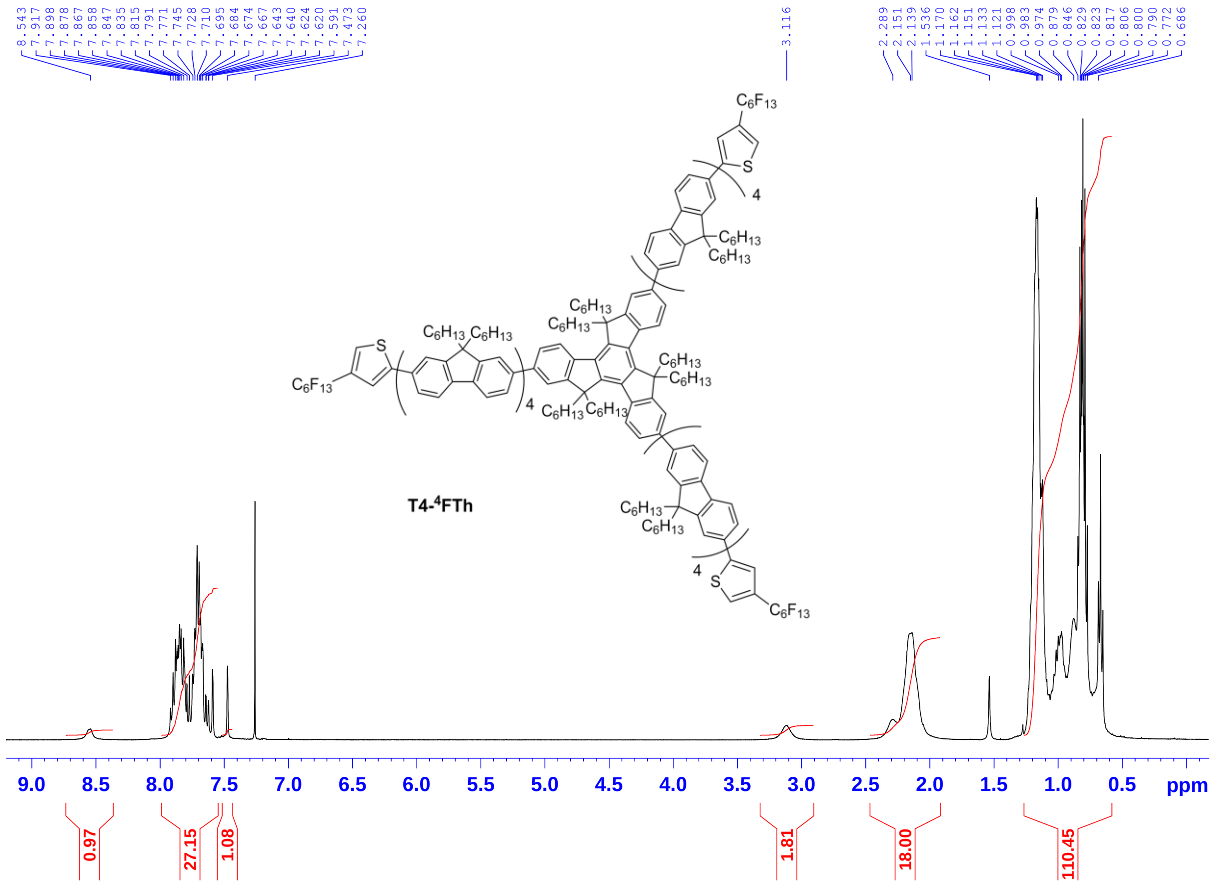

Figure S9: ^{1}H NMR of **T4-^{4}FTh**.

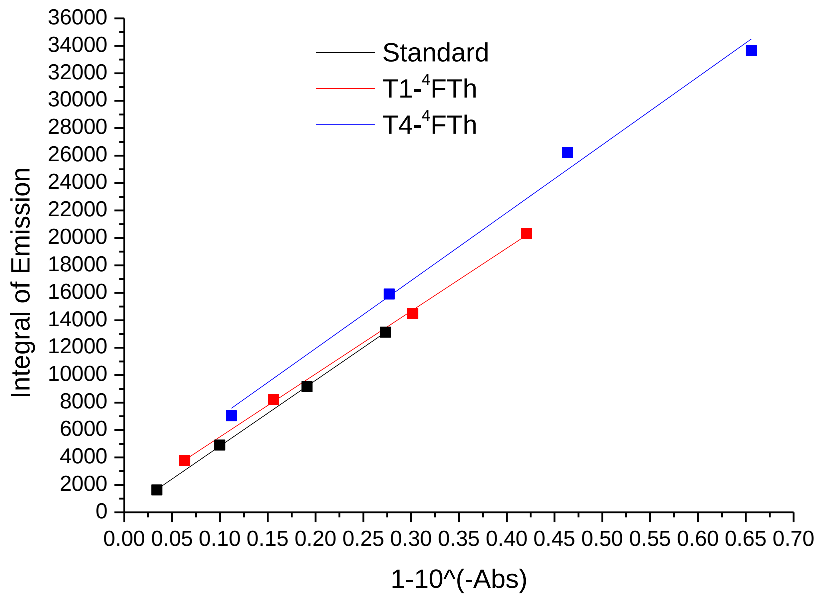

Figure S10: Plot of Integrated Emission Intensity vs Absorptance. 9,10-Diphenylanthracene was used as the Standard.

References

1. Wu, X.; Chen, T-A.; Zhu, L.; Rieke, R. D. *Tetrahedron. Lett.* **1994**, *35*, 363-3674*.*
2. Leroy, J.; Rubinstein, M.; Wakselman, C. *J. Fluor. Chem.* **1985**, *27*, 291-298.
3. Devarie-Baez, N. O.; Shuhler, B. J.; Wang, H.; Xian, M. *Org. Lett.* **2007**, *9*, 4655-4658.
